# The First Report of Cod Gill Poxvirus in Gills of Atlantic Cod ( *Gadus morhua* L.) Suffering From Cardiorespiratory Disease

**DOI:** 10.1111/jfd.14078

**Published:** 2024-12-27

**Authors:** Mona C. Gjessing, Torstein Tengs, Hanne Nilsen, Saima Mohammad, Simon C. Weli

**Affiliations:** ^1^ Department of Fish Health and Biosecurity Norwegian Veterinary Institute Oslo Norway; ^2^ Department of Breeding and Genetics Nofima Ås Norway

**Keywords:** *Ca*. Branchiomonas cysticola, emerging diseases, gill disease, gill poxvirus

## Abstract

Atlantic cod farming experiences renewed growth in Norway, and increased awareness is essential to address emerging diseases in this species. There are few reports on gill diseases in cod, and to date, no viral gill infections of cod have been documented. In this study, we collected samples from three sequential time points in summer 2023 from farmed cod suffering from cardiorespiratory disease. We document severe heart and gill pathology, and a novel double‐strand DNA virus was discovered in the gills. Through comprehensive genetic characterisation and comparative sequence analysis, this virus was classified as a new species in the genus Poxvirus, designated cod gill poxvirus (CGPV). We demonstrate disease causality with severe gill lesions as shown by histopathology and RNA scope in situ hybridisation, and poxvirus particles were identified in gill epithelial cells by transmission electron microscopy. Further, another gill pathogen not previously described in cod, *Candidatus*. Branchiomonas cysticola, was identified by pcr and in situ hybridisation. Our findings provide strong evidence for poxvirus in Atlantic cod and underscore the imminent threat posed to Atlantic cod farm industry.

## 1 Introduction

Atlantic cod ( *Gadus morhua* ), hereafter cod, is an economically important aquaculture fish species around the North Atlantic. The attempt at commercial cod farming around the turn of the millennium was partially successful, but production collapsed around 2012. Important reasons for this were various infectious diseases like Francisellosis caused by the bacterium *Francisella noatunensis* subsb. *noatunensis* (Mikalsen et al. 2007) access to other whitefish and early sexual maturation of the farmed cod (Sommerset et al. 2023). Now, cod‐farming is again growing in Norway, but diseases still cause problems with intestinal volvolus, spawning problems, circulatory disturbances and infectious diseases reported to be a great concerns (Sommerset et al. 2023). There are few viral diseases in cod, but viral nervous necrosis (VNN) caused by viral nervous necrosis virus (VNNV) can lead to high mortality rates, particularly among larvae and juvenile marine fish, including cod, as reviewed by Bandín and Souto (2020). The virus was isolated in farmed cod in Norway, the first time in 2007 (Patel et al. 2007) and can lead to severe pathological changes in central nervous system also in apparently healthy Atlantic cod (Gjessing et al. 2009). Another virus, infectious pancreas necrosis virus (IPNV) that can lead to infectious pancreatic necrosis (IPN) has so far only been described in two disease outbreaks in cod; one in Denmark and the other in Faroe Islands (Lorenzen et al. 1995). Further, infection with viral haemorrhagic septicaemia virus (VHSV) in wild cod has been described and experimental infection has been performed, but no clinical disease was reported (Snow et al. 2005). Nevertheless, due to the selection pressure in intensive fish farming and the tendency of RNA viruses to exhibit high mutation rates, more aggressive variants may emerge in these environments. Also, emergence of previously undescribed or novel diseases, or even the occurrence of several diseases in combination, is not uncommon in industrial fish farming. For example, in Atlantic salmon ( *Salmo salar* ) farming industry, virus‐related heart diseases such as, heart and skeletal inflammation (HSMI) (Kongtorp et al. 2004) seen in fish infected with piscine reovirus (PRV) and cardiomyopathy syndrome (CMS) (Fritsvold et al. 2009) caused by piscine myocarditis virus, that can be associated to mortalities. When these heart diseases are seen in combination with compromised gills disease, severe cardiorespiratory distress is often observed (personal observation). Apart from the microsporidium *Loma morhua* (Brown, Kent, and Adamson 2010) and the parasitic copepod *Lernaeocera branchialis* (Khan 1988), there are few reports on gill infections or gill infestations in cod. In Atlantic salmon, however, gill diseases are one of the major problems (Sommerset et al. 2023). Causes of gill diseases include non‐pathogenic (Rodger, Henry, and Mitchell 2011) and pathogenic agents (Gjessing et al. 2021). Gill disease caused by *Paramoeba perurans*, amoebic gill disease (AGD), the microsporidian *Desmozoon lepeoptherii*, bacteria *Ca*. Branchiomonas cysticola and salmon gill poxvirus (SGPV) have been well described (Gjessing et al. 2021). SGPV was discovered in 1995 (Kvellestad, unpublished results) and named by Nylund et al. (2008). The first fish poxvirus genome was sequenced in 2015 where SGPV was proven to be the phylogenetically deepest representative of the *Chordopoxvirinae* (Gjessing et al. 2015). The most apparent disease manifestation caused by SGPV, salmon gill poxvirus disease (SGPVD) can be observed in the freshwater phase with acute, high mortality (up to 70%) (Gjessing et al. 2017, 2020; Thoen et al. 2020). Salmon gill poxvirus infection, often in combination with other gill diseases, has been observed in salmon across various life stages, from fry to brood fish (Gjessing et al. 2017, 2021). Broad application of SGPV diagnostic techniques has revealed that SGPV can contribute to complex gill disease (CGD) in multifactorial infections in both freshwater and seawater farms (Gjessing et al. 2017).

Carp oedema virus (CEV), is another fish poxvirus that was discovered in Japan in 1974 (Ono, Nagai, and Sugai 1986), affecting carp ( *Cyprinus carpio* ). Infection with CEV can lead to swollen gills with hyperplasia of lamellar epithelium causing compromised respiratory and osmoregulatory function (Oyamatsu et al. 1997). CEV and SGPV appear to be highly host‐specific pathogens that primarily affect common carp and Atlantic salmon, respectively.

Descriptions of poxvirus infections in fish are relatively rare compared to other viral infections. The majority of well‐documented cases are in salmon and carp species. However, as diagnostic techniques improve, more cases may emerge in a wider range of species, especially in intensive aquaculture environments where fish health is closely monitored.

Here, we describe a cardiorespiratory disease outbreak in farmed Atlantic cod where a novel poxvirus, cod gill poxvirus (CGPV) was detected in the gills by next generation sequencing (NGS), PCR in situ hybridisation (ISH) and transmission electron microscopy (TEM). Although *Ca*. B. cysticola also was detected, histology, together with ISH demonstrates that CGPV can cause severe gill disease.

## 2 Methods

### 2.1 Fish Samples

The first fish sampling (referred to as sampling #I) was collected in mid‐May 2023, from a Norwegian cod farm in Western Norway, which experienced increased mortality. Additional fish sampling was performed in mid‐June (referred to as sampling #II) and in mid‐July (referred to as sampling #III) (Table 1). Fish were killed with an overdose of anaesthesia, and necropsy was performed. Gills, heart, liver, spleen and kidney samples were transferred to neutral phosphate‐buffered 10% formalin for histology and RNA scope in situ hybridisation. Separately, gills, brain and kidney were transferred to RNAlater (Qiagen Inc., Valencia, CA, USA) and stored at −80°C until used. Bacteriological cultivation was performed on kidney and on skin lesions.

**TABLE 1:** Overview of sampling time and type of material and methods used in the study.

Time of sampling/sampling id	Histology	Analysis for agents by PCR				Bacteriology
	Gill, heart, spleen, kidney, liver	BC, DL, SGPV, PP, CGPV	VHSV	IPNV	VNNV	Kidney
		Gill	Kidney	Kidney	Brain	
May 2023 /I	5	5	5	0	5	5^a^
June 2023/II	15	15	10	0	0	15^b^
July 2023/III	10	10	0	10	5	10^b^

Abbreviations: BC, *Ca*. Branchiomonas cysticola; CGPV, Cod gill poxvirus; DL, *Desmozoon lepeoptherii*; IPNV, Infectious pancreas necrosis virus; PP, Paramoeba perurans; SGPV, salmon gill poxvirus; VHSV, viral haemorrhagic septicaemia virus; VNNV, Viral nervous necrosis virus.

^a^ Blood agar with salt.

^b^ Blood agar with salt and cysteine heart agar plates supplemented with 5% blood.

### 2.2 Sequencing, PCR Design and Phylogenetic Analysis

Total RNA was isolated from 2 gill samples and 5 heart samples using the RNAeasy kit (QIAGEN) without on‐column DNase treatment. RNA was eluted using RNAse‐free water and used as template for the QuantiTect Whole Transcriptome Kit (Qiagen). Reverses transcription and whole transcriptome amplification were done using the recommended protocol, and resulting cDNA which was purified using ethanol precipitation. 1/10 volume of 3 M Na‐Acetate pH 5.2 and 2.5 volumes of ice‐cold 100% ethanol were used for each cDNA sample. The precipitated DNA was recovered by centrifugation at full speed in a microcentrifuge for 15–20 min, 4°C. The pellet was washed twice with room‐temperature 70% ethanol. Pellet was air dried and resuspended in a suitable volume of distilled water.

Reverse transcribed and amplified RNA was prepared for sequencing using the ThruPLEX DNA‐seq Kit (Takara Bio Europe) and paired‐end (2 × 150 bp) Illumina sequenced at the Norwegian Sequencing Centre (https://www.sequencing.uio.no/). Reads from host (Atlantic cod) were removed using bioinformatics procedures described by Tengs and Rimstad (2017). Briefly, all reads were trimmed using Trimmomatic (Bolger, Lohse, and Usadel 2014) (version 0.39) with recommended settings. Sets of trimmed reads were aligned with the cod genome and transcriptome (Star et al. 2011) (gadMor3.0) using bwa mem (Li and Durbin 2009) (version 0.7.17) with default parameters. Unmapped reads were extracted using SAMtools (Danecek et al. 2021) (version 1.18). Unmapped reads from the different samples were combined to make one set of paired‐end fastq files. These reads were *de novo* assembled using SPAdes (Prjibelski et al. 2020) (version 3.15.5) with the ‘—meta’ option and default parameters.

Resulting contigs were compared with all available protein data from salmon gill poxvirus (SGPV) (Gjessing et al. 2015) available in NCBI's GenBank using blastx (Altschul et al. 1990) (version 2.14.1) with default parameters.

Real‐time TaqMan PCR assay was designed using Primer3Plus (Untergasser et al. 2012) (version 3.3.0). The assay designed to detect our candidate virus sequence (cod_pox_F: TGACTATCTCCGAAAGAATCCC, cod_pox_R: ACGAACCTTCCCATCTTCC and cod_pox_taqman: CAGAAGGGAACGATTTCTAAGATAGTCCCAGACCA; FAM as 5′ reporter dye and TAMRA as 3′ quencher) (written in 5′‐3′ direction) was performed in 20 μL reactions containing 0,8 μM each primer, 0.4 μM probe, 2× Brilliant III UF (Agilent Technologies) and the final volume was adjusted with nuclease‐free water. Cycling conditions on an AriaMx Real‐time PCR system (Agilent) were 3 min denaturation at 95°C, followed by 45 cycles at 95°C for 10s and 60°C for 30 s.

Phylogenetic analyses were done using the program MEGA (Tamura, Stecher, and Kumar 2021) (version 11.0.13). Sequences were aligned using the implemented ClustalW algorithm, and a neighbour‐joining tree was made using the Poisson model with tree robustness tested using bootstrapping (1000 pseudoreplicates).

### 2.3 Agent Analysis: PCR and Bacteriology

Gills from all fish were analysed by PCR for CGPV (as described above) and for SGPV, *Ca*. B. Cysticola, D. lepeoptherii, P. perurans using the multiplex PCR described by Gjessing et al. (2021) (Table 1). From a subset of fish, brain was analysed by PCR for VNNV based on Baud et al. (2015) detecting most, known genotypes of the piscine nodavirus. Kidney was analysed by PCR for IPN based on Ørpetveit et al. (2010) and by PCR for VHSV detecting all known VHSV genotypes (Jonstrup et al. 2013) (Table 1). Bacteriological samples from kidney were streaked onto heart infusion agar containing 5% ovine blood (BA) supplemented with 1.5% NaCl. If fin or skin lesions were present, marine agar was included. The plates were incubated at 15°C and examined after 2, 4 and 6 days.

For sampling II and III, a subset of samples, on cysteine heart agar supplemented with 5% blood (CHAB) was incubated at 22°C to rule out the presence of *Francisella noatunensis* subsb. *noatunensis*.

### 2.4 Histology and In Situ Hybridisation

Haematoxylin and eosin (H&E)‐stained sections were made for histology. For a subset of the gills, in situ hybridisation analysis (ISH) was performed on serial sections to confirm the presence and to assess the distribution and localisation of agents detected using qPCR. Paired double‐Z oligonucleotide probes (V‐ACPV, Cat No. 1570041‐C1) were used to target CGPV L1R mRNA and both unique and conserved sequence regions of the *Ca*. B. cysticola genome (Acc. No. JN968376, RNAscope Cat No. 812001) as previously described (Gjessing et al. 2021). All CGPV negative PCR gill sections were included to assess the specificity of the probe and probe targeting cod ribosomal protein S20 mRNA (Gmor‐rps20‐C1, 1239771‐C1). Accession number: (GenBank XM_030348882.1) served as positive control. Staining was performed following the manufacturer's instructions.

### 2.5 Transmission Electron Microscopy

Corresponding regions in paraffin‐embedded gill tissue, where CGPV staining was detected by RNA scope, were identified. Fragments from these regions were then excised using a razor blade and processed for TEM following the protocol described by Lighezan et al. (2009).

## 3 Results

Here, we document severe gill and heart pathology in diseased farmed cod. All samples analysed were negative by PCR for SGPV and P. perurans, VNNV, IPNV and VHSV. No bacteria associated with any known disease in Atlantic cod were identified in bacteriological cultivation from kidney. Hence, there was no detection of previously known disease agents that could fully explain the lesions. Given the distinctive histopathological features observed, next‐generation sequencing (NGS) was the logical first step to investigate potential novel agents that could explain some of the histopathological lesions. The findings could guide the design of PCR and ISH assays as diagnostic tools for identifying any newly discovered agents.

### 3.1 Sequencing and PCR Design

**FIGURE 1:**
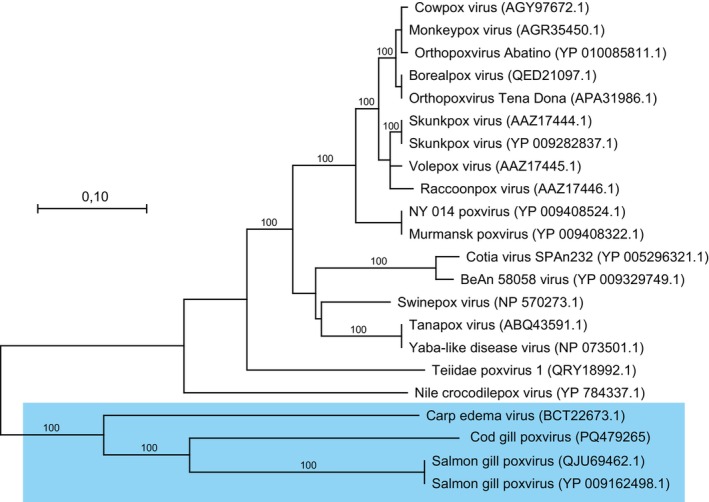
Phylogenetic tree for Cod gill poxvirus based on partial DNA‐directed RNA polymerase subunit beta gene (NJ), 1000 bootstrap pseudoreplicates.

Approximately 300 contigs produced by SPAdes had an e‐value below 0.0001 when using blastx to match the contigs with annotated protein from the SGPV genome (available upon request). Based on visual inspection of the highest‐scoring alignments, several contigs were identified that were explored further. A contig covering a part of the DNA‐directed RNA polymerase subunit beta gene (GenBank accession PQ479265) was used to perform a phylogenetic analysis (Figure 1) and used as target sequence for the PCR assay. In addition, a sequence with a high degree of similarity to the L1R gene in SGPV was identified (GenBank accession PQ479266), and this was chosen as a target for an in situ assay.

### 3.2 Disease Manifestation, Autopsy, Histopathology, In Situ Hybridisation

The cod were about 2 kg when increased mortality was reported in the farm and had lasted 4 days before sampling I (in May 2023). Camera images show that the fish suddenly turns and die. A number of jellyfish are observed in and around the cage, and CMS‐like pathology was reported at autopsy.

### 3.3 Heart Lesions

**FIGURE 2:**
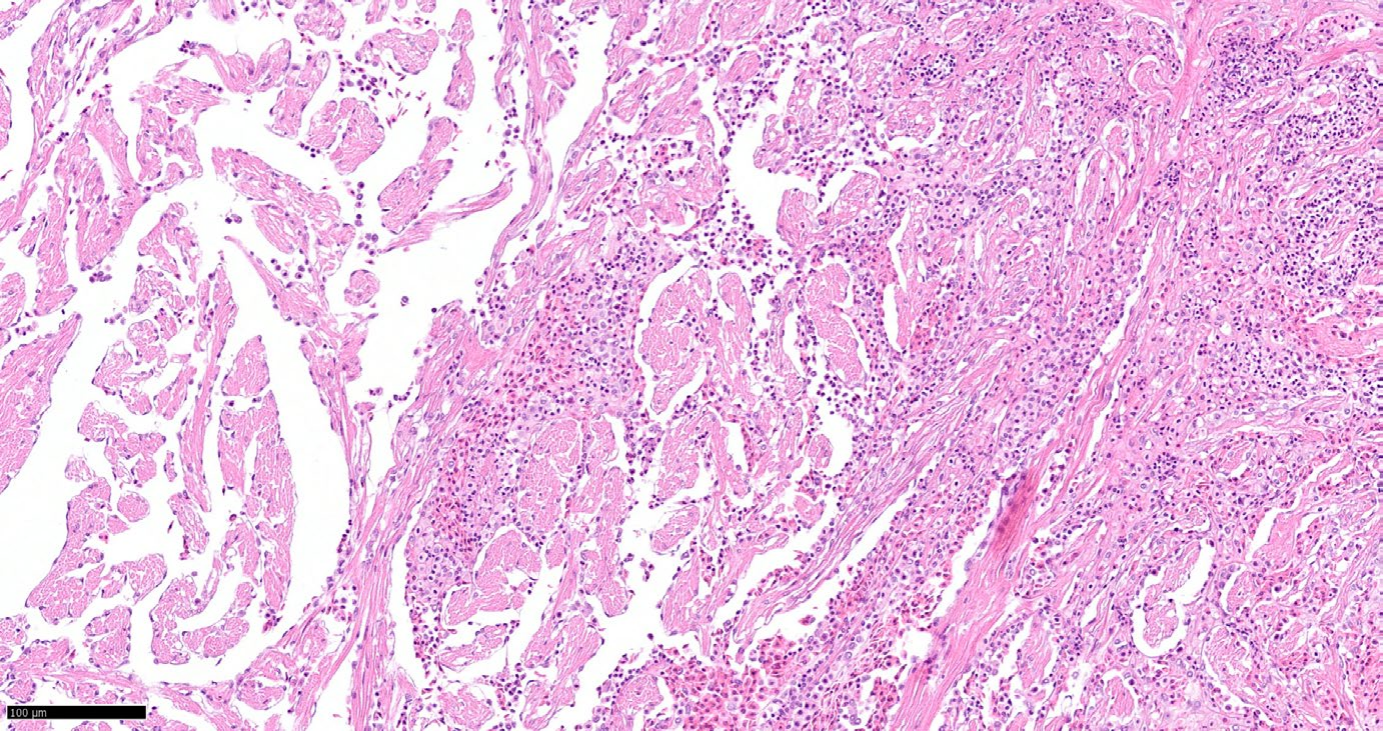
Section of Atlantic cod heart ventricle form sampling III. Presence of many inflammatory cells and circulatory disturbance Methods: HE‐stain.

Histopathological changes were seen in the heart with severe circulatory disturbance and foci of inflammatory cells. In sampling I few thrombi and small aggregations of inflammatory cells were seen in the cardiac lumen of some fish. In sampling II and III, the cardiac pathology was moderate to severe (Figure 2).

### 3.4 Gill Lesions

**FIGURE 3:**
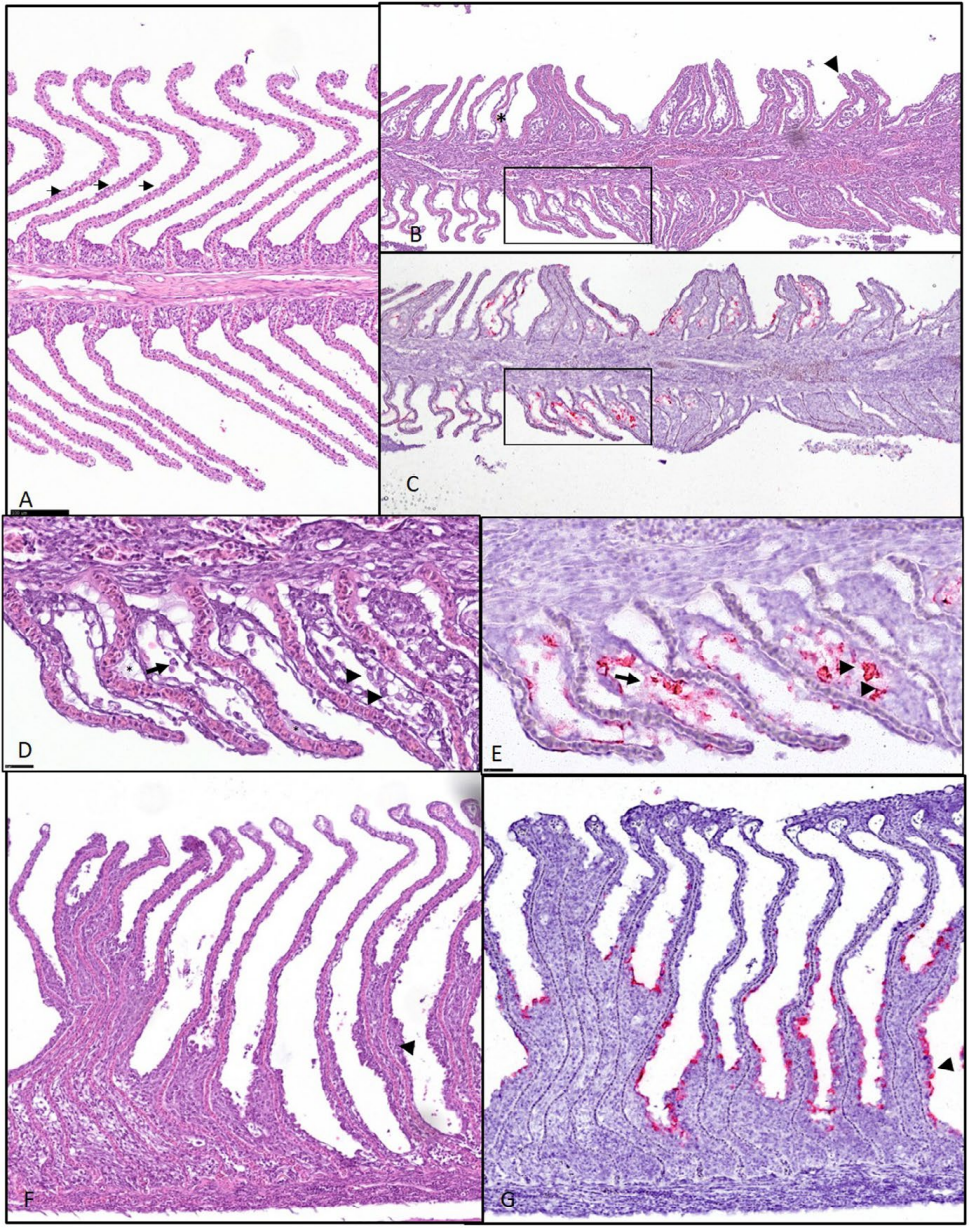
Sections of Atlantic cod gill (A) Normal gill. Note thin respiratory units (arrows) allowing efficient gas exchange. (B, C) Serial section of gill from a fish from sampling I. Lamellar adhesions (arrowheads), subepithelial oedema (asterisk). (C) Staining for CGPV (red) in epithelial cells covering lamella and interlamellar shedded epithelial cells. (D, E) Correspond to the area indicated in B and C respectively. (D, E) Note abnormal lamellar architecture with epithelial lifting and subepithelial oedema (asterisk) detached (arrows) and degenerated epithelial cells (arrowheads) staining for CGPV (red, arrowheads in E correspond to the area with arrowheads in D). (F) widespread lamellar adhesions (example indicated by arrowhead). (G) area correspond to F, demonstating extensive stainining for CGPV. Methods: HE‐stain (A, B, D, F) and RNA scope in situ hybridisation for cod gill poxvirus (CGPV) (C, E, G).

**FIGURE 4:**
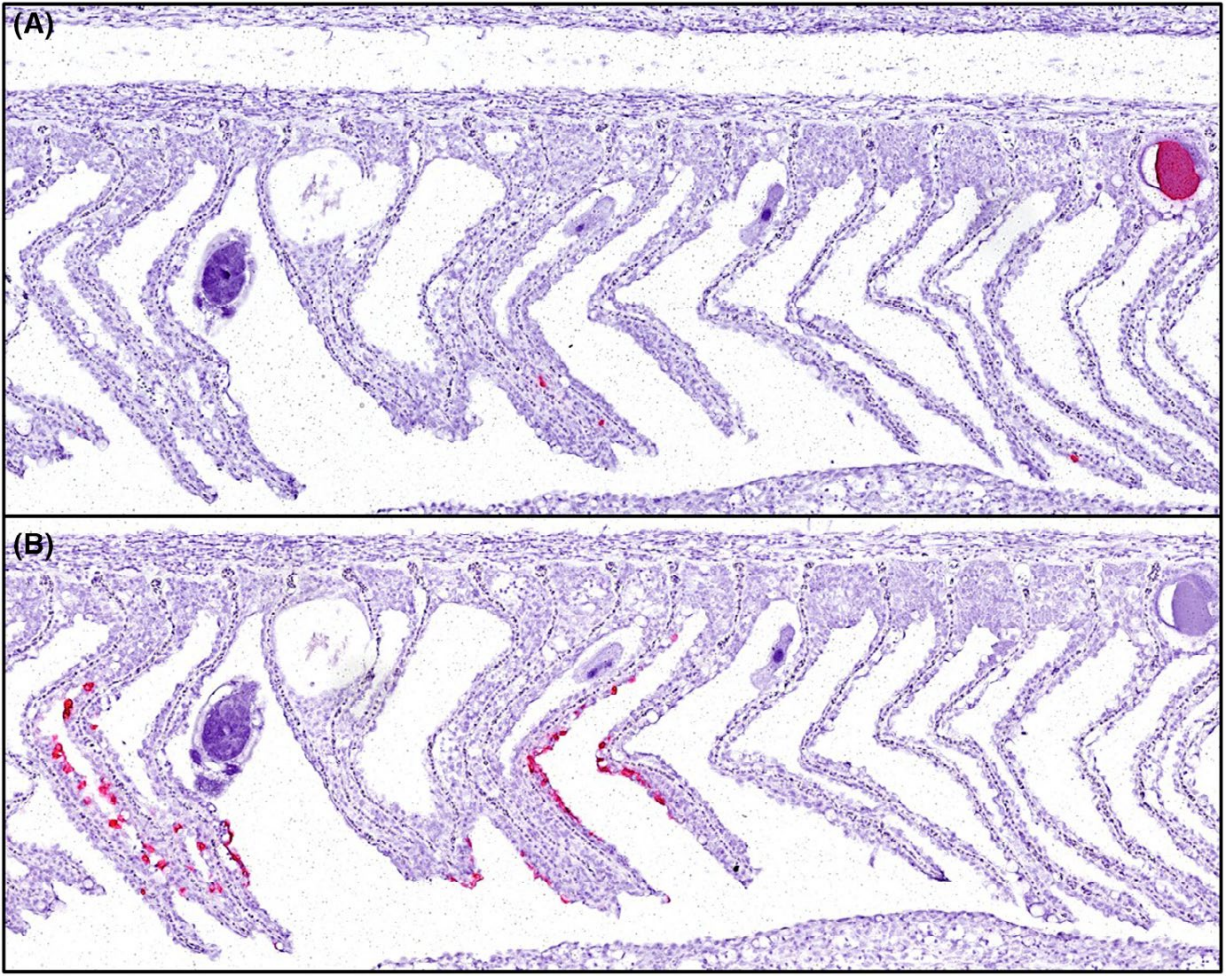
Sections of Atlantic cod gill. Methods: RNA scope in situ hybridisation for *Ca*. Branchiomonas cysticola (A) and cod gill poxvirus (CGPV) (B).

Gill histopathology for sampling I and II revealed extensive changes with many thickened lamellae (Figure 3B–D) due to hypertrophic and degeneration of respiratory epithelial cells (Figure 3D), many of which stained for CGPV by in situ hybridisation (Figure 3C,E). In many filaments, many lamellae were disorganised, and there were areas of lamellar adhesions with epithelial cells staining for CGPV and interlamellar cellular debris was seen (Figure 3D–G). Further, multifocal epithelial hyperplasia leading to coalescence of several lamellae where lacunae containing cells and debris were observed in some areas. Inflammatory cells were seen in parts of the gill filament in cod from sampling I and II. Epitheliocysts staining for *Ca*. B. cysticola was seen, but ISH also revealed the presence of *Ca*. B. cysticola within areas where no individual bacterial cells could be seen in H&E preparations (Figure 4A). *Trichkodina*‐like parasites were seen in some fish (Figure 4A,B). In sampling III, only sparse gill histopathology was seen.

Only minor findings were recorded in the spleen, kidney and liver and consisted of small granulomas suspected to be of minor clinical significance.

### 3.5 PCR and Transmission Electron Microscopy

PCR for CGPV on gill tissue demonstrated a significant difference in *C*_T_ values in the three different sampling timepoints with falling *C*_T_ values over time. The median *C*_T_ value was 24.4 (range from *C*_T_ 20.5 to *C*_T_ 27.1 *n* = 5) in sampling I, 28.5 (range from *C*_T_ 22.7 to *C*_T_ 35.5, *n* = 15) in sampling II and all fish were negative for CGPV in sampling III (*n* = 10). PCR for *Ca*. B. cysticola had *C*_T_ value with the median of 28.7 (range from *C*_T_ 26.3 to *C*_T_ 29.5 *n* = 5) in sampling I and a median of 26.2 (range from *C*_T_ 17.7 to *C*_T_ 34.3 *n* = 15) in sampling II and a median of 24 (range from *C*_T_ 21.8 to *C*_T_ 26.7 *n* = 10) in sampling III.

**FIGURE 5:**
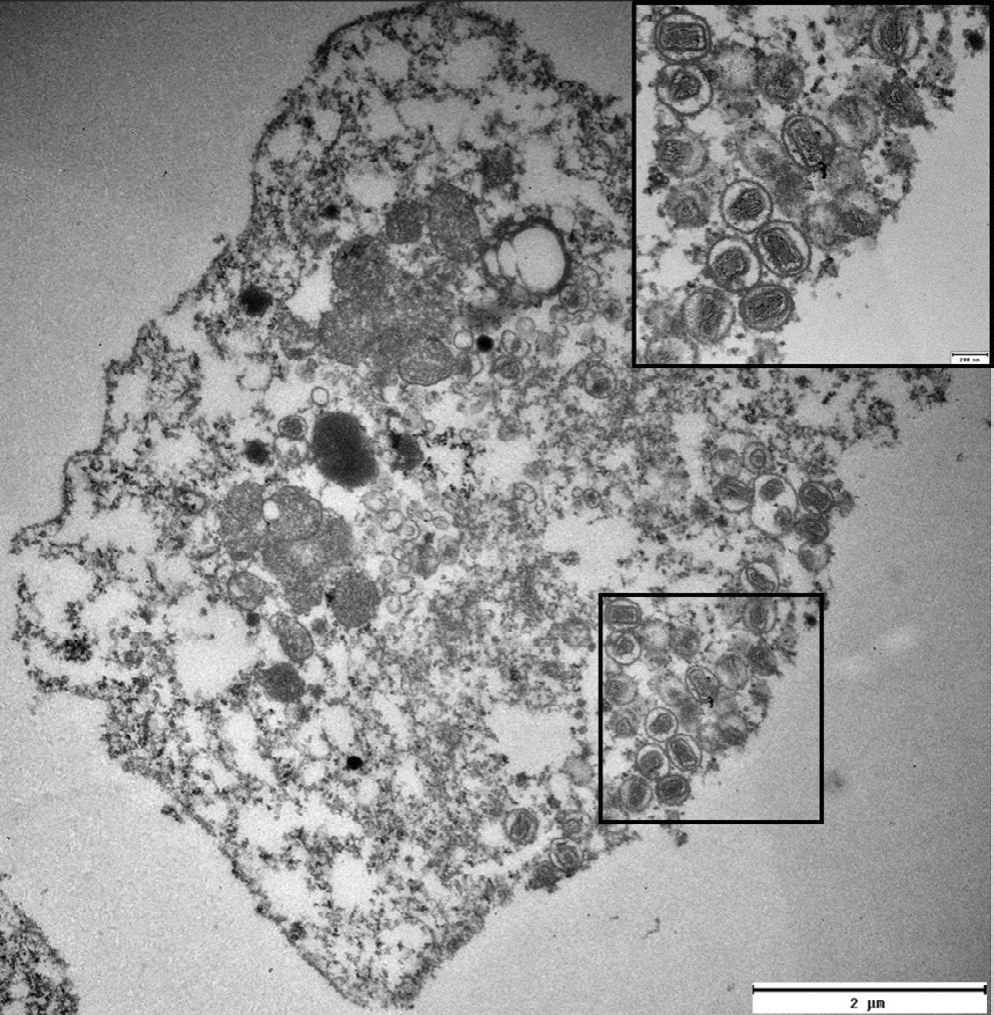
TEM showing virus particles consistent with poxvirus in size and shape in a gill epithelial cell, both in the cytoplasm and mature virus budding from the cell (insert).

From the gill from sampling I prepared for TEM, virus particles consistent with poxvirus in size and morphology with mature poxvirus particles were seen in epithelial cells. These were present in the cytoplasm or apparently budding from the cell (Figure 5).

### 3.6 Bacteriology

From sampling I, bacteriology culture revealed *Vibrio splendidus, Photobacterium* spp. and *Polaribacter* spp. from skin ulcers. From kidney in all samplings, no growth or very sparse growth of *Photobacterium* spp. and *Lactococcus* spp. from few fish. From one fish in sampling III, a pure culture of *Photobacterium iliopscarium* was seen and from one a pure culture of *Aliivibrio logei*.

## 4 Discussion

The emergence of viral pathogens from wild reservoirs poses significant threats to aquaculture fish health. Our study focused on investigating cardiorespiratory disease in farmed Atlantic cod that could jeopardise renewed growth in Norwegian cod farming. Through comprehensive Next generation sequencing analyses of Atlantic cod tissue samples collected from a cod farm at different time points, we successfully identified CGPV, a novel Poxvirus with the capacity to infect and cause histopathological changes in the gills of Atlantic cod. Thus, CGPV represents a third gill poxvirus that infects farmed fish, following the discovery of the first salmon gill poxvirus (SGPV) that infects Atlantic salmon (Gjessing et al. 2015) and CEV (Ono, Nagai, and Sugai 1986). Surprisingly, CGPV shows high sequence homologue to SGPV and is located in same clade in phylogenetic tree, suggesting CGPV may be a close relative of SGPV based on the DNA‐directed RNA polymerase subunit beta gene.

We successfully used TEM, qPCR, histopathology analysis, together with in situ hybridisation, to show that CGPV can cause severe gill histopathology in cod, a significant milestone in understanding CGPV disease pathogenesis and host interactions. Positive qPCR for CGPV and staining for CGPV in area with extensive gill histopathology was detected in fish collected in sampling I and II, but only sparse pathology when no virus was detected in sampling III. This suggests that CGPV is not merely an innocent bystander, but an agent that can cause severe gill disease in cod gills as seen in other poxvirus infections in fish, that is, Atlantic salmon suffering from SGPVD and carp suffering from KSD (Miyazaki, Isshiki, and Katsuyuki 2005). The combination of major blocking, due to epithelial cell hyperplasia and inflammation of the respiratory surfaces and cardiac pathology is a plausible explanation for the disease manifestation as both the respiratory and circulatory systems are essential for oxygen exchange and delivery to tissues, so their combined dysfunction has serious consequences. In Atlantic salmon, in situ hybridisation staining showed simple squamous lamellar epithelium of the gill was infected. The adjacent stratified epithelium showed no signs of infection, which suggests a narrow cell tropism. In Atlantic salmon affected by SGPVD, apoptotic gill epithelial cells were prominent and easily identifiable. In contrast, no distinct apoptotic gill cells were observed in the cod samples examined in this study. However, further investigation using special staining techniques and gene expression analysis is needed to confirm any lack of apoptotic cells. Gene expression analysis has recently being used to confirm lack of apoptotic gill epithelial cells in histopathology of carp suffering from KSD (Zawisza et al. 2024).

DNA viruses often form long‐standing, co‐evolved relationships with their hosts and tend to have more complex life cycles more integrated into their host's cellular machinery, making them more specialised and host‐specific. This host specificity is driven by the viruses' adaptation to their host's unique biological characteristics and environmental conditions that favour their proliferation in particular fish species (Koonin and Krupovic 2022). Atlantic cod, Atlantic salmon and carp are evolutionary very distinct groups of ray‐finned fish with carp even further apart from both salmon and cod. They have adapted to very different ecological niches and their significant evolutionary divergence suggests that if a poxvirus were adapted to one of these species, it would likely be highly specialised for that host due to the distinct physiological and ecological differences and very unlikely to cross over between distant species (McFadden 2005). *Ca*. B. cysticola was identified not only in the form of epitheliocysts, as expected, but also in some small foci, initially undetectable in H&E sections, as also reported in Atlantic salmon (Gjessing et al. 2021). Although CGPV appears to be responsible for most of the disease changes in the gills, *Ca*. B. cysticola and *trichodina* sp. have also been important contributors. The bacterial growth from ulcers and kidneys is presumed to be incidental findings, which did not contribute to the disease.

In summary, we identified CGPV, a novel gill poxvirus capable of infecting and causing disease in farmed Atlantic cod, marking it as the third known gill poxvirus affecting aquaculture fish. CGPV, closely related to the salmon gill poxvirus (SGPV), causes severe gill pathology, impacting the respiratory and circulatory systems critical for cod health. While *Ca*. B. cysticola and *trichodina* sp. were also present, CGPV appears to be the primary pathogen responsible for these disease manifestations. Our findings emphasise the importance of understanding host‐specific viral adaptations in fish, providing essential insights for monitoring and managing gill disease in cod farming.

The characterisation of CGPV advances our understanding of gill diseases in aquaculture species, providing crucial insights into pathogen diversity and fish health management. These findings carry important implications for managing viral disease outbreaks in cod farming, highlighting the need for monitoring and targeted interventions to protect the health and viability of farmed Atlantic cod populations.

## Author Contributions

**Mona C. Gjessing:** conceptualization, investigation, funding acquisition, writing – original draft, methodology, validation, visualization, writing – review and editing, project administration, formal analysis, data curation, supervision. **Torstein Tengs:** investigation, writing – original draft, data curation, methodology, software, formal analysis. **Hanne Nilsen:** validation, visualization, writing – review and editing. **Saima Mohammad:** methodology, writing – review and editing. **Simon C. Weli:** writing – review and editing, data curation.

## Conflicts of Interest

The authors declare no conflicts of interest.

## Supporting information


Table S1.

## Data Availability

GenBank accession PQ479265 and PQ479266.
